# Supplementary material for: Effectiveness of SARS-CoV-2 vaccines against Omicron infection and severe events: a systematic review and meta-analysis of test-negative design studies
Source: Front Public Health. 2023 Jun 9;11:1195908. doi: 10.3389/fpubh.2023.1195908 (PMC10289159; doi:10.3389/fpubh.2023.1195908)
Supplement: Supplementary file 1 [file Data_Sheet_1.PDF]

Supplementary Material

Effectiveness of SARS-CoV-2 Vaccines against Omicron Infection and Severe Events: A Systematic Review and Meta-Analysis of Test-Negative Design Studies

Shangchen Song, Zachary J. Madewell, Mingjin Liu, Ira M. Longini, Yang Yang

* **Correspondence:** Yang Yang Email: yang.yang4@uga.edu

Supplementary Materials..1
Supplementary Tables..2
Supplementary Figures..3
Searching Strategies..16

Supplementary Figure 1 Short-term vaccine effectiveness of full dose against infection or symptomatic infection..3
Supplementary Figure 2 Long-term vaccine effectiveness of full dose against infection or symptomatic infection..4
Supplementary Figure 3 Short-term vaccine effectiveness of first booster dose against infection or symptomatic infection..5
Supplementary Figure 4 Long-term vaccine effectiveness of first booster dose against infection or symptomatic infection..6
Supplementary Figure 5 Short-term vaccine effectiveness of full dose against Severe Events..7
Supplementary Figure 6 Long-term vaccine effectiveness of full dose against Severe Events..8
Supplementary Figure 7 Short-term and long-term vaccine effectiveness of first booster dose against Severe Events..9
Supplementary Figure 8 Funnel Plots of Main Analyses, Overall..11
Supplementary Figure 9 Funnel Plots of Main Analyses, Short Term..12
Supplementary Figure 10 Funnel Plots of Main Analyses, Long Term..13
Supplementary Figure 11 Funnel Plots of Main Analyses, Second Booster..14
Supplementary Figure 12 Funnel Plots of Subgroup Analyses that Show Publication Bias..15

Supplementary Tables

Supplementary Table 1 PICOS Criteria

Participant	The general population excluding disease-specific cohorts.
Intervention	Full doses, booster or second booster COVID-19 vaccinations
Comparator	Unvaccinated participants
Outcome	VE=1-adjusted odds ratio estimated in a (conditional) logistic regression
Study Type	Test-negative design study

Supplementary Figure 1 Short-term vaccine effectiveness of full dose against infection or symptomatic infection

Author(s) and Year	Region	Vaccine	Case Event	Age Group (years)	Test Timing (days)	Estimate [95% CI]
Pure mRNA Vaccines, full doses						
Florentino, et al. (2022)	Scotland	BNT162b2/BNT162b2	SI	12–17	14–97	0·648 [0·623, 0·671]
Powell, et al. (2021)	UK	BNT162b2/BNT162b2	SI	16–17	14–69	0·629 [0·610, 0·646]
Chemaitelly, et al. (2022)	Qatar	BNT162b2/BNT162b2	SI	all ages	30–119	0·478 [0·408, 0·539]
Kim, et al. (2022)	USA	Mixed (BNT162b2/mRNA–1273)	SI	≥18	14–149	0·450 [0·140, 0·660]
Tseng, et al. (2022a)	USA	mRNA–1273/mRNA–1273	AI	≥18	14–90	0·440 [0·351, 0·516]
Chemaitelly, et al. (2022)	Qatar	mRNA–1273/mRNA–1273	SI	all ages	30–119	0·432 [0·150, 0·621]
Andrews, et al. (2022)	UK	mRNA–1273/mRNA–1273	SI	≥18	14–104	0·423 [0·400, 0·445]
Andrews, et al. (2022)	UK	BNT162b2/BNT162b2	SI	≥18	14–104	0·410 [0·400, 0·419]
Buchan, et al. (2022b)	Canada	BNT162b2/BNT162b2	SI	12–17	7–119	0·378 [0·293, 0·452]
Fleming–Dutra, et al. (2022)	USA	BNT162b2/BNT162b2	SI	5–11	(14–30) & (60–90)	0·362 [0·326, 0·396]
Florentino, et al. (2022)	Brazil	BNT162b2/BNT162b2	SI	12–17	14–97	0·341 [0·332, 0·351]
Lind, et al. (2022)	Brazil	Mixed (BNT162b2/mRNA–1273)	AI	≥5	14–149	0·285 [0·200, 0·362]
Fleming–Dutra, et al. (2022)	USA	BNT162b2/BNT162b2	SI	12–15	(14–30) & (60–90)	0·215 [0·139, 0·285]
Pooled VE in adults: (Q = 1·72, df = 3, p = 0·63; I^2 = 9·3%, τ^2 = 6·50e–05)						0·413 [0·402, 0·424]
Pooled VE in children and adolescents: (Q = 781·29, df = 5, p < ·01; I^2 = 99·4%, τ^2 = 1·09e–01)						0·453 [0·287, 0·581]
Pooled VE in all age groups: (Q = 826·53, df = 12, p < ·01; I^2 = 99·2%, τ^2 = 5·48e–02)						0·435 [0·354, 0·506]
Partial mRNA Vaccines, full doses						
Kirsebom, et al. (2022a) BA2	UK	Mixed (BNT162b2/mRNA–1273/ChAdOx1)	SI	≥18	14–175	0·449 [0·431, 0·466]
Andeweg, et al. (2022) BA2	Netherlands	Mixed (BNT162b2/mRNA–1273/ChAdOx1)	SI	≥18	30–119	0·430 [0·351, 0·499]
Andeweg, et al. (2022) BA1	Netherlands	Mixed (BNT162b2/mRNA–1273/ChAdOx1)	SI	≥18	14–119	0·412 [0·359, 0·460]
Kirsebom, et al. (2022a) BA1	UK	Mixed (BNT162b2/mRNA–1273/ChAdOx1)	SI	≥18	14–175	0·349 [0·334, 0·365]
Braeye, et al. (2022)	Belgium	Mixed (BNT162b2/mRNA–1273/ChAdOx1)	SI	≥18	0–100	0·208 [0·194, 0·221]
Buchan, et al. (2022a)	Canada	Mixed (≥1 mRNA Vaccine)	SI	≥18	7–119	0·197 [0·124, 0·264]
Pooled VE in adults: (Q = 488·76, df = 5, p < ·01; I^2 = 98·8%, τ^2 = 2·65e–02)						0·347 [0·254, 0·429]
Pooled VE in adults across all vaccine types: (Q = 833·10, df = 9, p < ·01; I^2 = 98·6%, τ^2 = 1·97e–02)						0·375 [0·314, 0·431]
Pooled VE in all age groups across all vaccine types: (Q = 1666·46, df = 18, p < ·01; I^2 = 99·3%, τ^2 = 4·84e–02)						0·407 [0·343, 0·465]
Test for VE Difference in All across Vaccine Types: Q_M = 1·74, df = 1, p = 0·19						

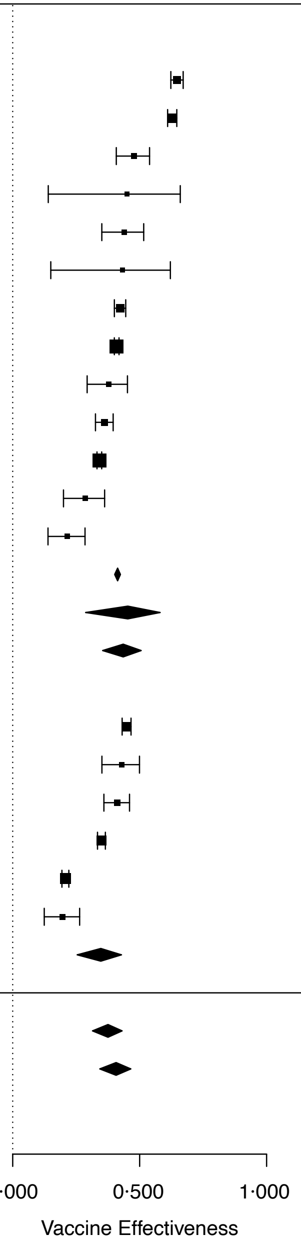

Supplementary Figure 2 Long-term vaccine effectiveness of full dose against infection or symptomatic infection

Author(s) and Year	Region	Vaccine	Case Event	Age Group (years)	Test Timing (days)	Estimate [95% CI]
Pure mRNA Vaccines, full doses						
Florentino, et al. (2022)	Scotland	BNT162b2/BNT162b2	SI	12–17	≥98	0·313 [0·048, 0·505]
Buchan, et al. (2022b)	Canada	BNT162b2/BNT162b2	SI	12–17	≥120	0·290 [0·216, 0·357]
Powell, et al. (2021)	UK	BNT162b2/BNT162b2	SI	16–17	≥70	0·226 [0·145, 0·299]
Chemaitelly, et al. (2022)	Qatar	mRNA–1273/mRNA–1273	SI	all ages	120–419	0·187 [0·113, 0·255]
Chemaitelly, et al. (2022)	Qatar	BNT162b2/BNT162b2	SI	all ages	120–419	0·163 [0·097, 0·225]
Lind, et al. (2022)	Brazil	Mixed (BNT162b2/mRNA–1273)	AI	≥5	≥150	0·153 [0·104, 0·200]
Andrews, et al. (2022)	UK	mRNA–1273/mRNA–1273	SI	≥18	≥140	0·150 [0·118, 0·181]
Florentino, et al. (2022)	Brazil	BNT162b2/BNT162b2	SI	12–17	≥98	0·139 [0·109, 0·169]
Tseng, et al. (2022a)	USA	mRNA–1273/mRNA–1273	AI	≥18	181–270	0·138 [0·102, 0·173]
Andrews, et al. (2022)	UK	BNT162b2/BNT162b2	SI	≥18	≥105	0·126 [0·118, 0·134]
Kim, et al. (2022)	USA	Mixed (BNT162b2/mRNA–1273)	SI	≥18	≥150	0·110 [−0·210, 0·350]
Pooled VE in adults: ($Q = 2{\cdot}44$, df = 3, $p = 0{\cdot}49$; $I^2 = 20{\cdot}4\%$, $\tau^2 = 8{\cdot}14e{-}05$)						0·131 [0·117, 0·146]
Pooled VE in children and adolescents: ($Q = 16{\cdot}98$, df = 3, $p < {\cdot}01$; $I^2 = 78{\cdot}7\%$, $\tau^2 = 7{\cdot}94e{-}03$)						0·223 [0·136, 0·301]
Pooled VE in all age groups: ($Q = 30{\cdot}71$, df = 10, $p < {\cdot}01$; $I^2 = 77{\cdot}0\%$, $\tau^2 = 1{\cdot}66e{-}03$)						0·164 [0·136, 0·191]
Partial mRNA Vaccines, full doses						
Andeweg, et al. (2022) BA2	Netherlands	Mixed (BNT162b2/mRNA–1273/ChAdOx1)	SI	≥18	150–209	0·337 [0·299, 0·373]
Andeweg, et al. (2022) BA1	Netherlands	Mixed (BNT162b2/mRNA–1273/ChAdOx1)	SI	≥18	150–209	0·301 [0·274, 0·327]
Kirsebom, et al. (2022a) BA2	UK	Mixed (BNT162b2/mRNA–1273/ChAdOx1)	SI	≥18	≥175	0·278 [0·259, 0·297]
Kirsebom, et al. (2022a) BA1	UK	Mixed (BNT162b2/mRNA–1273/ChAdOx1)	SI	≥18	≥175	0·148 [0·129, 0·167]
Buchan, et al. (2022a)	Canada	Mixed (≥1 mRNA Vaccine)	SI	≥18	180–239	0·010 [−0·080, 0·100]
Pooled VE in adults: ($Q = 183{\cdot}87$, df = 4, $p < {\cdot}01$; $I^2 = 98{\cdot}7\%$, $\tau^2 = 2{\cdot}51e{-}02$)						0·226 [0·108, 0·327]
Non–mRNA Vaccines, full doses						
Ranzani, et al. (2022)	Brazil	CoronaVac/CoronaVac	SI	≥75	≥180	0·281 [0·247, 0·313]
Ranzani, et al. (2022)	Brazil	CoronaVac/CoronaVac	SI	60–74	≥180	0·220 [0·198, 0·242]
Kirsebom, et al. (2022b)	UK	ChAdOx1/ChAdOx1	SI	≥65	≥175	0·195 [0·117, 0·266]
Kirsebom, et al. (2022b)	UK	ChAdOx1/ChAdOx1	SI	40–64	≥175	0·080 [0·060, 0·099]
Ranzani, et al. (2022)	Brazil	CoronaVac/CoronaVac	SI	18–59	≥180	0·008 [−0·004, 0·020]
Andrews, et al. (2022)	UK	ChAdOx1/ChAdOx1	SI	≥18	≥175	−0·027 [−0·042, −0·012]
Pooled VE in adults: ($Q = 500{\cdot}14$, df = 5, $p < {\cdot}01$; $I^2 = 99{\cdot}4\%$, $\tau^2 = 2{\cdot}02e{-}02$)						0·132 [0·026, 0·226]
Pooled VE in adults across all vaccine types: ($Q = 1399{\cdot}28$, df = 14, $p < {\cdot}01$; $I^2 = 99{\cdot}2\%$, $\tau^2 = 1{\cdot}80e{-}02$)						0·166 [0·105, 0·223]
Pooled VE in all age groups across all vaccine types: ($Q = 1448{\cdot}84$, df = 21, $p < {\cdot}01$; $I^2 = 98{\cdot}6\%$, $\tau^2 = 1{\cdot}38e{-}02$)						0·176 [0·132, 0·218]
Test for VE Difference in All across Vaccine Types: $Q_M = 2{\cdot}76$, df = 2, $p = 0{\cdot}25$						

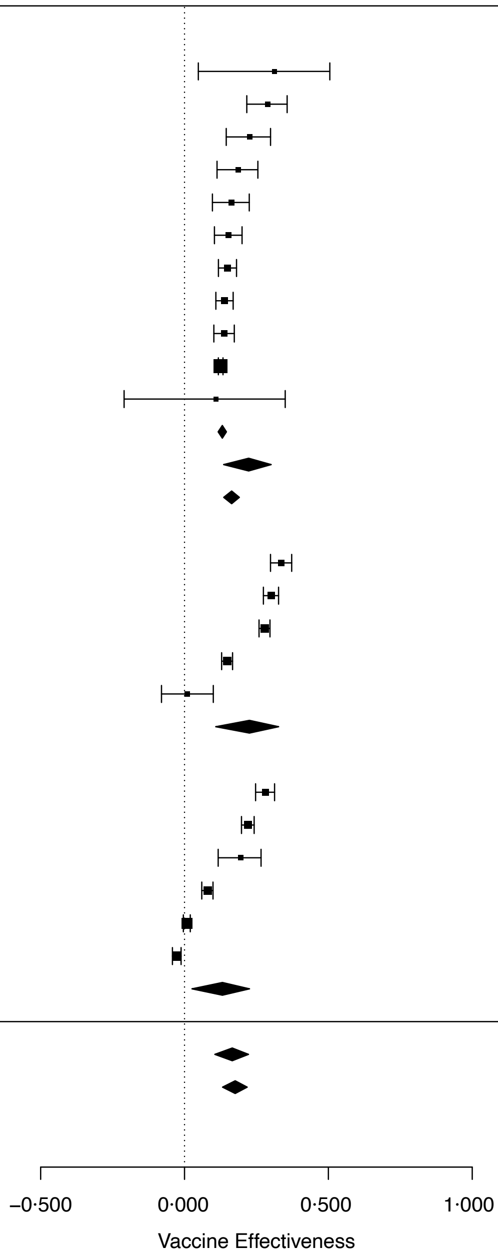

Supplementary Figure 3 Short-term vaccine effectiveness of first booster dose against infection or symptomatic infection

Author(s) and Year	Region	Vaccine	Case Event	Age Group (years)	Test Timing (days)	Estimate [95% CI]
Pure mRNA Vaccines, booster dose						
Andrews, et al. (2022)	UK	BNT162b2/BNT162b2/mRNA−1273	SI	≥18	7−69	0·725 [0·720, 0·731]
Accorsi, et al. (2022)	USA	mRNA−1273/mRNA−1273/mRNA−1273	SI	≥18	14−90	0·720 [0·690, 0·740]
Tseng, et al. (2022a)	USA	mRNA−1273/mRNA−1273/mRNA−1273	AI	≥18	14−60	0·716 [0·697, 0·734]
Fleming−Dutra, et al. (2022)	USA	BNT162b2/BNT162b2/BNT162b2	SI	12−15	14−45	0·711 [0·655, 0·757]
Andrews, et al. (2022)	UK	mRNA−1273/mRNA−1273/mRNA−1273	SI	≥18	7−34	0·672 [0·654, 0·689]
Accorsi, et al. (2022)	USA	Mixed (BNT162b2/mRNA−1273)	SI	≥18	14−90	0·670 [0·650, 0·690]
Accorsi, et al. (2022)	USA	BNT162b2/BNT162b2/BNT162b2	SI	≥18	14−90	0·650 [0·620, 0·680]
Andrews, et al. (2022)	UK	mRNA−1273/mRNA−1273/BNT162b2	SI	≥18	7−34	0·646 [0·628, 0·663]
Andrews, et al. (2022)	UK	BNT162b2/BNT162b2/BNT162b2	SI	≥18	7−69	0·625 [0·621, 0·630]
Grewal, et al. (2022a)	Canada	Mixed (BNT162b2/mRNA−1273)	SI	≥60	<84	0·620 [0·510, 0·710]
Altarawneh, et al. (2022)	Qatar	mRNA−1273/mRNA−1273/mRNA−1273	SI	all ages	7−60	0·613 [0·533, 0·679]
Grewal, et al. (2022a)	Canada	BNT162b2/BNT162b2/mRNA−1273	SI	≥60	20−110	0·570 [0·400, 0·690]
Altarawneh, et al. (2022)	Qatar	BNT162b2/BNT162b2/BNT162b2	SI	all ages	7−60	0·540 [0·504, 0·573]
Chemaitelly, et al. (2022)	Qatar	mRNA−1273/mRNA−1273/mRNA−1273	SI	all ages	7−41	0·471 [0·384, 0·546]
Chemaitelly, et al. (2022)	Qatar	BNT162b2/BNT162b2/BNT162b2	SI	all ages	7−69	0·440 [0·405, 0·473]
Pooled VE in adults: ($Q = 756{\cdot}16$, df = 9, $p < {\cdot}01$; $I^2 = 97{\cdot}4\%$, $\tau^2 = 1{\cdot}44e{-}02$)						0·673 [0·645, 0·699]
Pooled VE in all age groups: ($Q = 1083{\cdot}00$, df = 14, $p < {\cdot}01$; $I^2 = 98{\cdot}9\%$, $\tau^2 = 4{\cdot}82e{-}02$)						0·637 [0·592, 0·677]
Partial mRNA Vaccines, booster dose						
Andeweg, et al. (2022) BA1	Netherlands	Mixed (BNT162b2/mRNA−1273/ChAdOx1)	SI	≥18	7−59	0·690 [0·682, 0·698]
Kirsebom, et al. (2022a) BA2	UK	Mixed (BNT162b2/mRNA−1273/ChAdOx1)	SI	≥18	7−69	0·683 [0·676, 0·690]
Andrews, et al. (2022)	UK	ChAdOx1/ChAdOx1/mRNA−1273	SI	≥18	7−69	0·677 [0·672, 0·682]
Kirsebom, et al. (2022a) BA1	UK	Mixed (BNT162b2/mRNA−1273/ChAdOx1)	SI	≥18	7−69	0·660 [0·655, 0·665]
Andeweg, et al. (2022) BA2	Netherlands	Mixed (BNT162b2/mRNA−1273/ChAdOx1)	SI	≥18	7−59	0·634 [0·616, 0·650]
Ranzani, et al. (2022)	Brazil	CoronaVac/CoronaVac/BNT162b2	SI	≥75	8−59	0·625 [0·602, 0·647]
Kirsebom, et al. (2022b)	UK	ChAdOx1/ChAdOx1/BNT162b2	SI	≥65	7−69	0·606 [0·585, 0·627]
Kirsebom, et al. (2022b)	UK	ChAdOx1/ChAdOx1/BNT162b2	SI	40−64	7−69	0·602 [0·597, 0·607]
Andrews, et al. (2022)	UK	ChAdOx1/ChAdOx1/BNT162b2	SI	≥18	7−69	0·583 [0·579, 0·588]
Ranzani, et al. (2022)	Brazil	CoronaVac/CoronaVac/BNT162b2	SI	60−74	8−59	0·579 [0·565, 0·593]
Ranzani, et al. (2022)	Brazil	CoronaVac/CoronaVac/BNT162b2	SI	18−59	8−59	0·573 [0·568, 0·579]
Braeye, et al. (2022)	Belgium	Mixed (BNT162b2/mRNA−1273/ChAdOx1)	SI	≥18	0−50	0·520 [0·520, 0·530]
Pooled VE in adults: ($Q = 3423{\cdot}84$, df = 11, $p < {\cdot}01$; $I^2 = 99{\cdot}6\%$, $\tau^2 = 1{\cdot}86e{-}02$)						0·623 [0·592, 0·651]
Non−mRNA Vaccines, booster dose						
Kirsebom, et al. (2022b)	UK	ChAdOx1/ChAdOx1/ChAdOx1	SI	40−64	7−69	0·536 [0·464, 0·598]
Andrews, et al. (2022)	UK	ChAdOx1/ChAdOx1/ChAdOx1	SI	≥18	7−69	0·520 [0·446, 0·583]
Kirsebom, et al. (2022b)	UK	ChAdOx1/ChAdOx1/ChAdOx1	SI	≥65	7−69	0·489 [0·334, 0·607]
Ranzani, et al. (2022)	Brazil	CoronaVac/CoronaVac/CoronaVac	SI	≥75	8−59	0·371 [0·267, 0·460]
Ranzani, et al. (2022)	Brazil	CoronaVac/CoronaVac/CoronaVac	SI	60−74	8−59	0·122 [0·047, 0·190]
Ranzani, et al. (2022)	Brazil	CoronaVac/CoronaVac/CoronaVac	SI	18−59	8−59	0·081 [0·046, 0·114]
Pooled VE in adults: ($Q = 175{\cdot}64$, df = 5, $p < {\cdot}01$; $I^2 = 97{\cdot}2\%$, $\tau^2 = 9{\cdot}08e{-}02$)						0·372 [0·195, 0·510]
Pooled VE in adults across all vaccine types: ($Q = 7437{\cdot}23$, df = 27, $p < {\cdot}01$; $I^2 = 99{\cdot}8\%$, $\tau^2 = 8{\cdot}76e{-}02$)						0·599 [0·551, 0·641]
Pooled VE in all age groups across all vaccine types: ($Q = 7614{\cdot}05$, df = 32, $p < {\cdot}01$; $I^2 = 99{\cdot}8\%$, $\tau^2 = 8{\cdot}33e{-}02$)						0·594 [0·551, 0·633]
Test for VE Difference in All across Vaccine Types: $Q_M = 30{\cdot}00$, df = 2, p = 0·00						

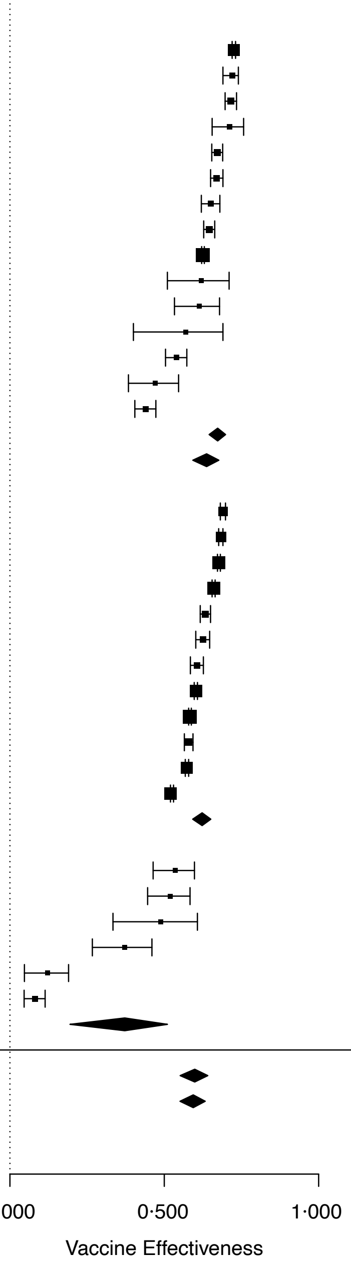

Supplementary Figure 4 Long-term vaccine effectiveness of first booster dose against infection or symptomatic infection

Author(s) and Year	Region	Vaccine	Case Event	Age Group (years)	Test Timing (days)	Estimate [95% CI]
Pure mRNA Vaccines, booster dose						
Grewal, et al. (2022a)	Canada	mRNA−1273/mRNA−1273/mRNA−1273	SI	≥60	72−152	0·610 [0·500, 0·690]
Grewal, et al. (2022a)	Canada	Mixed (BNT162b2/mRNA−1273)	SI	≥60	≥84	0·550 [0·450, 0·640]
Grewal, et al. (2022a)	Canada	BNT162b2/BNT162b2/BNT162b2	SI	≥60	70−145	0·530 [0·390, 0·630]
Tseng, et al. (2022a)	USA	mRNA−1273/mRNA−1273/mRNA−1273	AI	≥18	>60	0·474 [0·405, 0·535]
Andrews, et al. (2022)	UK	BNT162b2/BNT162b2/BNT162b2	SI	≥18	≥70	0·457 [0·447, 0·467]
Chemaitelly, et al. (2022)	Qatar	mRNA−1273/mRNA−1273/mRNA−1273	SI	all ages	≥42	0·349 [0·146, 0·504]
Chemaitelly, et al. (2022)	Qatar	BNT162b2/BNT162b2/BNT162b2	SI	all ages	≥98	0·219 [0·077, 0·339]
Pooled VE in adults: ($Q = 11·66$, $df = 4$, $p = 0·02$; $I^2 = 69·7\%$, $\tau^2 = 1·01e−02$)						0·509 [0·450, 0·562]
Pooled VE in all age groups: ($Q = 31·84$, $df = 6$, $p < ·01$; $I^2 = 89·9\%$, $\tau^2 = 4·20e−02$)						0·466 [0·368, 0·548]
Partial mRNA Vaccines, booster dose						
Ranzani, et al. (2022)	Brazil	CoronaVac/CoronaVac/BNT162b2	SI	≥75	≥60	0·450 [0·427, 0·473]
Kirsebom, et al. (2022a) BA2	UK	Mixed (BNT162b2/mRNA−1273/ChAdOx1)	SI	≥18	≥105	0·437 [0·423, 0·451]
Andeweg, et al. (2022) BA1	Netherlands	Mixed (BNT162b2/mRNA−1273/ChAdOx1)	SI	≥18	90−119	0·430 [0·350, 0·500]
Ranzani, et al. (2022)	Brazil	CoronaVac/CoronaVac/BNT162b2	SI	60−74	≥60	0·418 [0·403, 0·433]
Andrews, et al. (2022)	UK	ChAdOx1/ChAdOx1/BNT162b2	SI	≥18	≥70	0·396 [0·380, 0·411]
Kirsebom, et al. (2022a) BA1	UK	Mixed (BNT162b2/mRNA−1273/ChAdOx1)	SI	≥18	≥105	0·374 [0·358, 0·390]
Ranzani, et al. (2022)	Brazil	CoronaVac/CoronaVac/BNT162b2	SI	18−59	≥60	0·318 [0·311, 0·325]
Kirsebom, et al. (2022b)	UK	ChAdOx1/ChAdOx1/BNT162b2	SI	40−64	≥105	0·306 [0·268, 0·343]
Braeye, et al. (2022)	Belgium	Mixed (BNT162b2/mRNA−1273/ChAdOx1)	SI	≥18	100−150	0·250 [0·240, 0·270]
Kirsebom, et al. (2022b)	UK	ChAdOx1/ChAdOx1/BNT162b2	SI	≥65	≥105	0·231 [0·151, 0·305]
Andeweg, et al. (2022) BA2	Netherlands	Mixed (BNT162b2/mRNA−1273/ChAdOx1)	SI	≥18	90−149	0·129 [0·115, 0·144]
Pooled VE in adults: ($Q = 1545·77$, $df = 10$, $p < ·01$; $I^2 = 99·3\%$, $\tau^2 = 2·19e−02$)						0·346 [0·286, 0·402]
Non−mRNA Vaccines, booster dose						
Kirsebom, et al. (2022b)	UK	ChAdOx1/ChAdOx1/ChAdOx1	SI	40−64	≥105	0·372 [−0·441, 0·726]
Ranzani, et al. (2022)	Brazil	CoronaVac/CoronaVac/CoronaVac	SI	≥75	≥60	0·132 [0·088, 0·175]
Ranzani, et al. (2022)	Brazil	CoronaVac/CoronaVac/CoronaVac	SI	60−74	≥60	0·067 [0·027, 0·105]
Ranzani, et al. (2022)	Brazil	CoronaVac/CoronaVac/CoronaVac	SI	18−59	≥60	−0·105 [−0·151, −0·057]
Pooled VE in adults: ($Q = 59·14$, $df = 3$, $p < ·01$; $I^2 = 95·0\%$, $\tau^2 = 1·48e−02$)						0·046 [−0·095, 0·169]
Pooled VE in adults across all vaccine types: ($Q = 3043·85$, $df = 22$, $p < ·01$; $I^2 = 99·7\%$, $\tau^2 = 8·19e−02$)						0·315 [0·227, 0·394]
Pooled VE in all age groups across all vaccine types: ($Q = 3000·28$, $df = 21$, $p < ·01$; $I^2 = 99·6\%$, $\tau^2 = 5·91e−02$)						0·349 [0·276, 0·415]
Test for VE Difference in All across Vaccine Types: $Q_M = 24·94$, $df = 2$, $p = 0·00$						

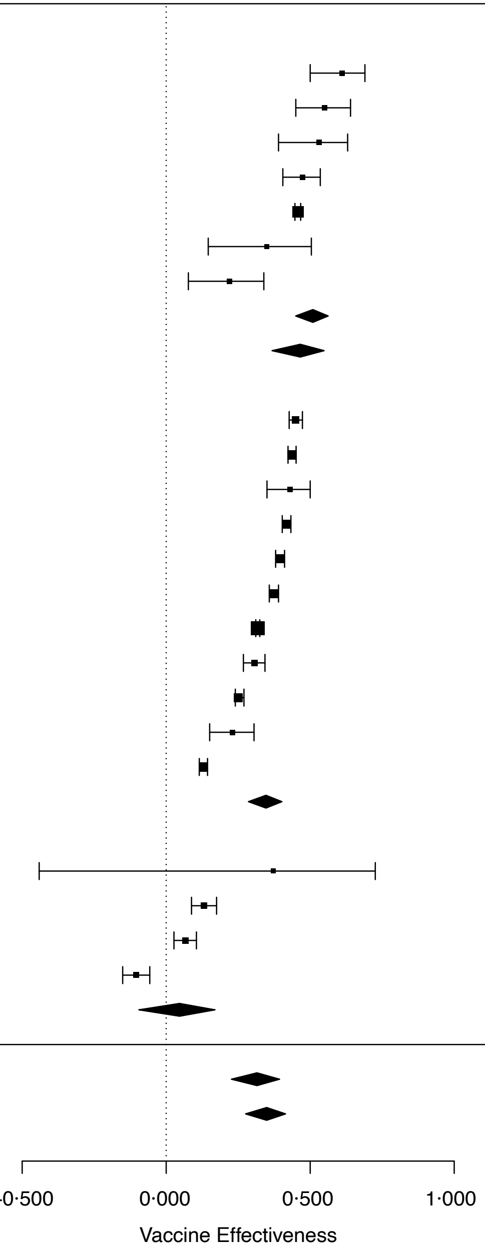

Supplementary Figure 5 Short-term vaccine effectiveness of full dose against Severe Events

Author(s) and Year	Region	Vaccine	Case Event	Age Group (years)	Test Timing (days)	Estimate [95% CI]
Pure mRNA Vaccines, full doses						
Florentino, et al. (2022)	Brazil	BNT162b2/BNT162b2	H/D	12–17	14–97	0·826 [0·793, 0·854]
Buchan, et al. (2022b)	Canada	BNT162b2/BNT162b2	H/D	12–17	7–119	0·814 [0·587, 0·916]
Ferdinands, et al. (2022a)	USA	Mixed (BNT162b2/mRNA−1273)	H	≥18	<60	0·710 [0·510, 0·830]
Gary, et al. (2022)	South Africa	BNT162b2/BNT162b2	H	≥18	14–87	0·709 [0·644, 0·761]
Tartof, et al. (2022a)	USA	BNT162b2/BNT162b2	H	≥18	<90	0·680 [0·480, 0·800]
Klein, et al. (2022)	USA	BNT162b2/BNT162b2	ED/UC	5–11	14–67	0·510 [0·300, 0·650]
Klein, et al. (2022)	USA	BNT162b2/BNT162b2	ED/UC	12–15	14–149	0·450 [0·300, 0·570]
Price, et al. (2022)	USA	BNT162b2/BNT162b2	H	12–18	14–160	0·430 [−0·010, 0·680]
Klein, et al. (2022)	USA	BNT162b2/BNT162b2	ED/UC	16–17	14–149	0·340 [0·080, 0·530]
Pooled VE in adults: ($Q = 0{\cdot}13$, $df = 2$, $p = 0{\cdot}94$; $I^2 = 0{\cdot}0\%$, $\tau^2 = 0{\cdot}00e{+}00$)						0·705 [0·649, 0·752]
Pooled VE in children and adolescents: ($Q = 94{\cdot}01$, $df = 5$, $p < {\cdot}01$; $I^2 = 92{\cdot}1\%$, $\tau^2 = 3{\cdot}06e{-}01$)						0·607 [0·366, 0·756]
Pooled VE in all age groups: ($Q = 94{\cdot}81$, $df = 8$, $p < {\cdot}01$; $I^2 = 88{\cdot}9\%$, $\tau^2 = 2{\cdot}00e{-}01$)						0·640 [0·502, 0·740]
Partial mRNA Vaccines, full doses						
Kirsebom, et al. (2022a) BA2	UK	Mixed (BNT162b2/mRNA−1273/ChAdOx1)	H	≥18	14–175	0·863 [0·528, 0·960]
Stowe, et al. (2022)	UK	Mixed (BNT162b2/mRNA−1273/ChAdOx1)	EC −> H	≥65	14–174	0·808 [0·641, 0·897]
Natarajan, et al. (2022)	USA	Mixed (Janssen/1mRNADose)	H	≥18	7–120	0·780 [0·700, 0·840]
Kirsebom, et al. (2022a) BA1	UK	Mixed (BNT162b2/mRNA−1273/ChAdOx1)	H	≥18	14–175	0·761 [0·593, 0·860]
Stowe, et al. (2022)	UK	Mixed (BNT162b2/mRNA−1273/ChAdOx1)	EC −> H	18–64	14–174	0·593 [0·539, 0·641]
Buchan, et al. (2022a)	Canada	Mixed (≥1 mRNA Vaccine)	H/D	≥18	7–59	0·550 [−1·060, 0·900]
Adams, et al. (2022)	USA	Mixed (BNT162b2/mRNA−1273/Janssen)	H	≥18	14–150	0·530 [0·330, 0·670]
Pooled VE in adults: ($Q = 23{\cdot}82$, $df = 6$, $p < {\cdot}01$; $I^2 = 75{\cdot}5\%$, $\tau^2 = 1{\cdot}16e{-}01$)						0·707 [0·592, 0·789]
Pooled VE in adults across all vaccine types: ($Q = 28{\cdot}24$, $df = 9$, $p < {\cdot}01$; $I^2 = 67{\cdot}1\%$, $\tau^2 = 5{\cdot}84e{-}02$)						0·699 [0·628, 0·756]
Pooled VE in all age groups across all vaccine types: ($Q = 123{\cdot}06$, $df = 15$, $p < {\cdot}01$; $I^2 = 87{\cdot}0\%$, $\tau^2 = 1{\cdot}63e{-}01$)						0·669 [0·583, 0·738]

Test for VE Difference in All across Vaccine Types: $Q_M = 0{\cdot}80$, $df = 1$, $p = 0{\cdot}37$

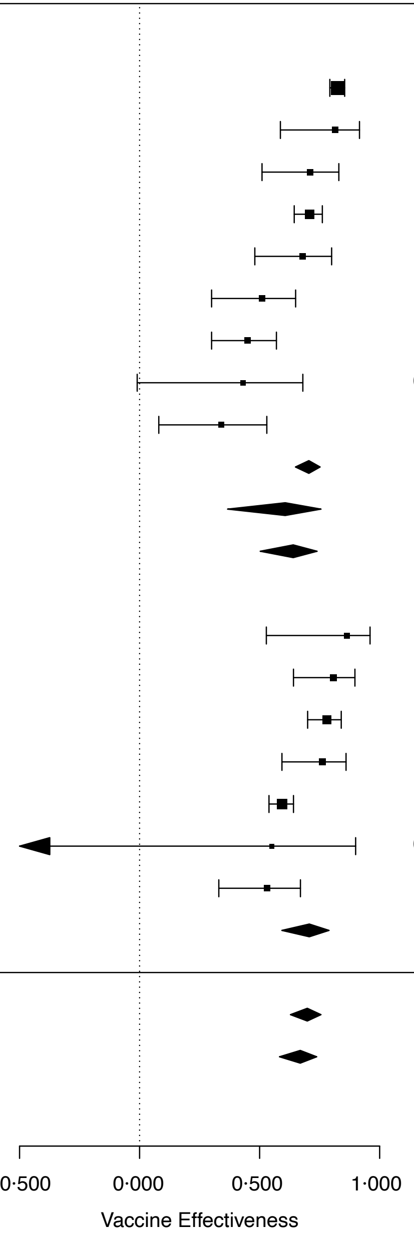

Supplementary Figure 6 Long-term vaccine effectiveness of full dose against Severe Events

Author(s) and Year	Region	Vaccine	Case Event	Age Group (years)	Test Timing (days)	Estimate [95% CI]
Pure mRNA Vaccines, full doses						
Buchan, et al. (2022b)	Canada	BNT162b2/BNT162b2	H/D	12–17	≥120	0·854 [0·763, 0·910]
Florentino, et al. (2022)	Brazil	BNT162b2/BNT162b2	H/D	12–17	≥98	0·849 [0·752, 0·908]
Tenforde, et al. (2022)	USA	Mixed (BNT162b2/mRNA–1273)	INV/D	≥18	>256	0·790 [0·660, 0·870]
Tartof, et al. (2022a)	USA	BNT162b2/BNT162b2	H	≥18	180–269	0·720 [0·630, 0·790]
Gary, et al. (2022)	South Africa	BNT162b2/BNT162b2	H	≥18	148–207	0·670 [0·630, 0·710]
Ferdinands, et al. (2022a)	USA	Mixed (BNT162b2/mRNA–1273)	H	≥18	≥150	0·540 [0·480, 0·590]
Price, et al. (2022)	USA	BNT162b2/BNT162b2	H	12–18	161–314	0·380 [–0·030, 0·620]
Klein, et al. (2022)	USA	BNT162b2/BNT162b2	ED/UC	12–15	≥150	–0·020 [–0·250, 0·170]
Klein, et al. (2022)	USA	BNT162b2/BNT162b2	ED/UC	16–17	≥150	–0·030 [–0·300, 0·180]
Pooled VE in adults: (Q = 25·26, df = 3, p < ·01; I^2 = 90·1%, τ^2 = 7·68e–02)						0·677 [0·563, 0·761]
Pooled VE in children and adolescents: (Q = 100·77, df = 4, p < ·01; I^2 = 97·1%, τ^2 = 9·33e–01)						0·564 [–0·036, 0·817]
Pooled VE in all age groups: (Q = 197·55, df = 8, p < ·01; I^2 = 97·5%, τ^2 = 5·18e–01)						0·624 [0·389, 0·768]
Partial mRNA Vaccines, full doses						
Buchan, et al. (2022a)	Canada	Mixed (≥1 mRNA Vaccine)	H/D	≥18	≥120	0·790 [0·663, 0·869]
Stowe, et al. (2022)	UK	Mixed (BNT162b2/mRNA–1273/ChAdOx1)	EC –> H	≥65	≥175	0·634 [0·505, 0·729]
Kirsebom, et al. (2022a) BA1	UK	Mixed (BNT162b2/mRNA–1273/ChAdOx1)	H	≥18	≥175	0·506 [0·377, 0·608]
Adams, et al. (2022)	USA	Mixed (BNT162b2/mRNA–1273/Janssen)	H	≥18	>150	0·340 [0·210, 0·450]
Stowe, et al. (2022)	UK	Mixed (BNT162b2/mRNA–1273/ChAdOx1)	EC –> H	18–64	≥175	0·334 [0·253, 0·406]
Kirsebom, et al. (2022a) BA2	UK	Mixed (BNT162b2/mRNA–1273/ChAdOx1)	H	≥18	≥175	0·259 [–0·111, 0·506]
Pooled VE in adults: (Q = 37·11, df = 5, p < ·01; I^2 = 92·7%, τ^2 = 1·69e–01)						0·507 [0·299, 0·652]
Non–mRNA Vaccines, full doses						
Ranzani, et al. (2022)	Brazil	CoronaVac/CoronaVac	H/D	18–59	≥180	0·710 [0·659, 0·753]
Ranzani, et al. (2022)	Brazil	CoronaVac/CoronaVac	H/D	60–74	≥180	0·634 [0·583, 0·679]
Ranzani, et al. (2022)	Brazil	CoronaVac/CoronaVac	H/D	≥75	≥180	0·407 [0·337, 0·469]
Pooled VE in adults: (Q = 60·91, df = 2, p < ·01; I^2 = 96·6%, τ^2 = 1·29e–01)						0·601 [0·397, 0·736]
Pooled VE in adults across all vaccine types: (Q = 187·41, df = 12, p < ·01; I^2 = 95·1%, τ^2 = 1·44e–01)						0·590 [0·490, 0·671]
Pooled VE in all age groups across all vaccine types: (Q = 332·11, df = 17, p < ·01; I^2 = 97·1%, τ^2 = 3·13e–01)						0·583 [0·455, 0·681]
Test for VE Difference in All across Vaccine Types: Q_M = 0·62, df = 2, p = 0·73						

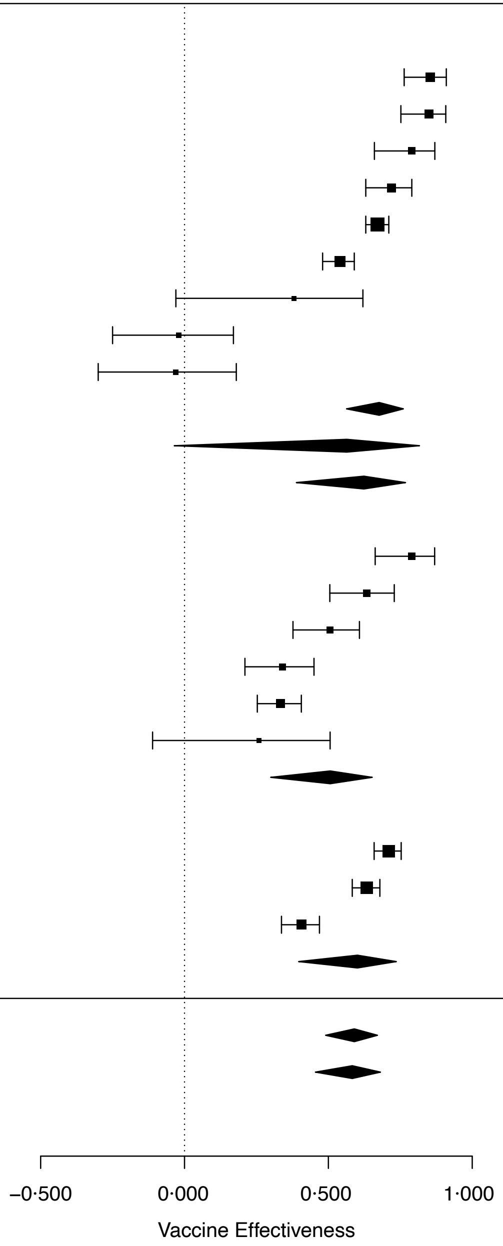

Supplementary Figure 7 Short-term and long-term vaccine effectiveness of first booster dose against Severe Events

Author(s) and Year	Region	Vaccine	Case Event	Age Group (years old)	Test Timing (days)	Estimate [95% CI]
Pure mRNA Vaccines, booster dose, short term						
Ferdinands, et al. (2022a)	USA	Mixed (BNT162b2/mRNA−1273)	H	≥18	<60	0·910 [0·880, 0·930]
Natarajan, et al. (2022)	USA	Mixed (BNT162b2/mRNA−1273)	H	≥18	7−120	0·900 [0·880, 0·910]
Tartof, et al. (2022a)	USA	BNT162b2/BNT162b2/BNT162b2	H	≥18	<90	0·850 [0·800, 0·890]
Grewal, et al. (2022a)	Canada	BNT162b2/BNT162b2/mRNA−1273	H/D	≥60	16−98	0·810 [0·670, 0·890]
Grewal, et al. (2022a)	Canada	Mixed (BNT162b2/mRNA−1273)	H/D	≥60	<84	0·800 [0·720, 0·860]
Grewal, et al. (2022a)	Canada	BNT162b2/BNT162b2/BNT162b2	H/D	≥60	24−112	0·770 [0·670, 0·830]
Pooled VE in adults: (Q = 38·43, df = 5, p < ·01; I^2 = 86·7%, τ^2 = 1·33e−01)						0·853 [0·798, 0·893]
Partial mRNA Vaccines, booster dose, short term						
Stowe, et al. (2022)	UK	Mixed (BNT162b2/mRNA−1273/ChAdOx1)	EC −> H	≥65	7−69	0·935 [0·919, 0·947]
Ranzani, et al. (2022)	Brazil	CoronaVac/CoronaVac/BNT162b2	H/D	18−59	8−59	0·921 [0·902, 0·937]
Kirsebom, et al. (2022a) BA1	UK	Mixed (BNT162b2/mRNA−1273/ChAdOx1)	H	≥18	7−69	0·892 [0·866, 0·913]
Ranzani, et al. (2022)	Brazil	CoronaVac/CoronaVac/BNT162b2	H/D	60−74	8−59	0·884 [0·862, 0·902]
Kirsebom, et al. (2022a) BA2	UK	Mixed (BNT162b2/mRNA−1273/ChAdOx1)	H	≥18	7−69	0·872 [0·786, 0·923]
Stowe, et al. (2022)	UK	Mixed (BNT162b2/mRNA−1273/ChAdOx1)	EC −> H	18−64	7−69	0·823 [0·807, 0·837]
Ranzani, et al. (2022)	Brazil	CoronaVac/CoronaVac/BNT162b2	H/D	≥75	8−59	0·773 [0·731, 0·808]
Pooled VE in adults: (Q = 142·46, df = 6, p < ·01; I^2 = 95·7%, τ^2 = 1·84e−01)						0·881 [0·834, 0·914]
Non−mRNA Vaccines, booster dose, short term						
Ranzani, et al. (2022)	Brazil	CoronaVac/CoronaVac/CoronaVac	H/D	18−59	8−59	0·860 [0·717, 0·931]
Ranzani, et al. (2022)	Brazil	CoronaVac/CoronaVac/CoronaVac	H/D	60−74	8−59	0·807 [0·686, 0·881]
Natarajan, et al. (2022)	USA	Janssen/Janssen	H	≥18	7−120	0·670 [0·520, 0·770]
Ranzani, et al. (2022)	Brazil	CoronaVac/CoronaVac/CoronaVac	H/D	≥75	8−59	0·477 [0·124, 0·688]
Pooled VE in adults: (Q = 12·29, df = 3, p < ·01; I^2 = 78·6%, τ^2 = 2·35e−01)						0·730 [0·537, 0·843]
Pure mRNA Vaccines, booster dose, long term						
Tenforde, et al. (2022)	USA	Mixed (BNT162b2/mRNA−1273)	INV/D	≥18	>60	0·940 [0·880, 0·970]
Grewal, et al. (2022a)	Canada	mRNA−1273/mRNA−1273/mRNA−1273	H/D	≥60	70−150	0·810 [0·740, 0·860]
Ferdinands, et al. (2022a)	USA	Mixed (BNT162b2/mRNA−1273)	H	≥18	120−149	0·780 [0·670, 0·850]
Grewal, et al. (2022a)	Canada	Mixed (BNT162b2/mRNA−1273)	H/D	≥60	≥84	0·770 [0·690, 0·820]
Tartof, et al. (2022a)	USA	BNT162b2/BNT162b2/BNT162b2	H	≥18	≥90	0·550 [0·280, 0·710]
Pooled VE in adults: (Q = 23·98, df = 4, p < ·01; I^2 = 91·1%, τ^2 = 3·85e−01)						0·801 [0·646, 0·888]
Partial mRNA Vaccines, booster dose, long term						
Ranzani, et al. (2022)	Brazil	CoronaVac/CoronaVac/BNT162b2	H/D	60−74	≥60	0·904 [0·891, 0·915]
Ranzani, et al. (2022)	Brazil	CoronaVac/CoronaVac/BNT162b2	H/D	18−59	≥60	0·902 [0·883, 0·917]
Stowe, et al. (2022)	UK	Mixed (BNT162b2/mRNA−1273/ChAdOx1)	EC −> H	≥65	≥105	0·862 [0·812, 0·898]
Kirsebom, et al. (2022a) BA1	UK	Mixed (BNT162b2/mRNA−1273/ChAdOx1)	H	≥18	≥105	0·804 [0·756, 0·843]
Ranzani, et al. (2022)	Brazil	CoronaVac/CoronaVac/BNT162b2	H/D	≥75	≥60	0·785 [0·761, 0·806]
Stowe, et al. (2022)	UK	Mixed (BNT162b2/mRNA−1273/ChAdOx1)	EC −> H	18−64	≥105	0·605 [0·512, 0·681]
Kirsebom, et al. (2022a) BA2	UK	Mixed (BNT162b2/mRNA−1273/ChAdOx1)	H	≥18	≥105	0·565 [0·384, 0·693]
Adams, et al. (2022)	USA	Mixed (BNT162b2/mRNA−1273/Janssen)	H	≥18	>120	0·390 [0·220, 0·530]
Pooled VE in adults: (Q = 327·30, df = 7, p < ·01; I^2 = 98·2%, τ^2 = 4·74e−01)						0·780 [0·643, 0·864]
Non−mRNA Vaccines, booster dose, long term						
Ranzani, et al. (2022)	Brazil	CoronaVac/CoronaVac/CoronaVac	H/D	18−59	≥60	0·820 [0·609, 0·917]
Ranzani, et al. (2022)	Brazil	CoronaVac/CoronaVac/CoronaVac	H/D	60−74	≥60	0·761 [0·708, 0·804]
Ranzani, et al. (2022)	Brazil	CoronaVac/CoronaVac/CoronaVac	H/D	≥75	≥60	0·514 [0·446, 0·573]
Pooled VE in adults: (Q = 37·97, df = 2, p < ·01; I^2 = 94·2%, τ^2 = 2·22e−01)						0·705 [0·473, 0·835]
Pooled VE in adults across all vaccine types, Short Term: (Q = 235·81, df = 16, p < ·01; I^2 = 94·9%, τ^2 = 2·45e−01)						0·848 [0·804, 0·881]
Pooled VE in adults across all vaccine types, Long Term: (Q = 539·10, df = 15, p < ·01; I^2 = 97·0%, τ^2 = 3·72e−01)						0·776 [0·694, 0·836]

Test for Short Term VE Difference in Adults across Vaccine Types: Q_M = 7·82, df = 2, p = 0·02
Test for Long Term VE Difference in Adults across Vaccine Types: Q_M = 0·57, df = 2, p = 0·75

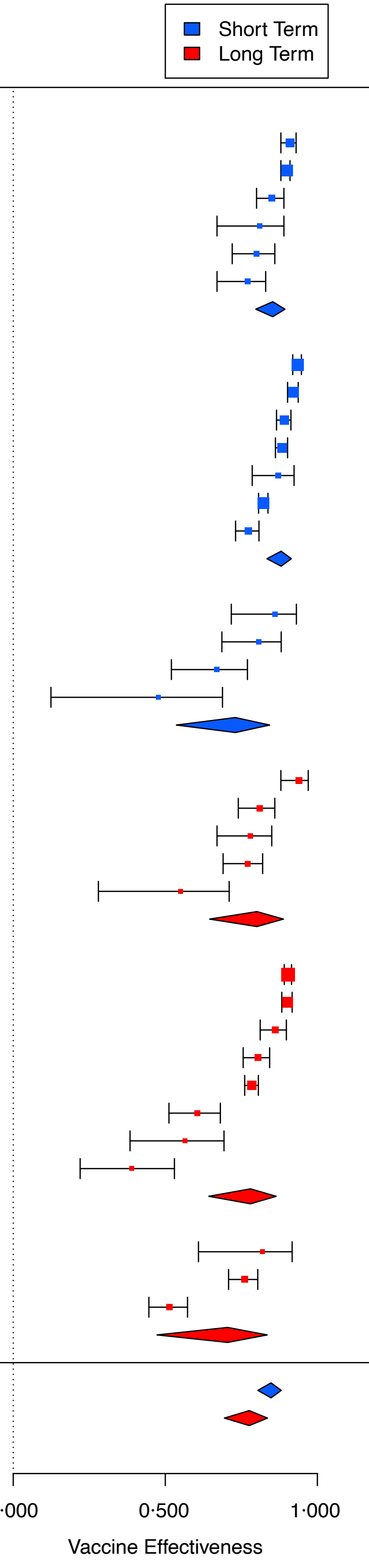

Acronyms in the Supplementary Figures:

SI: symptomatic infection

AI: all infection

D: death

H: hospitalization

ED/UC: emergency department (ED) or urgent care (UC) encounter

ED: emergency department admission

EC→H: hospital admissions from emergency care

H/D: hospitalization or death

ICU: intensive care unit (ICU) admission

NCH: noncritical hospitalization

INV/D: invasive mechanical ventilation/death

INV: invasive ventilation

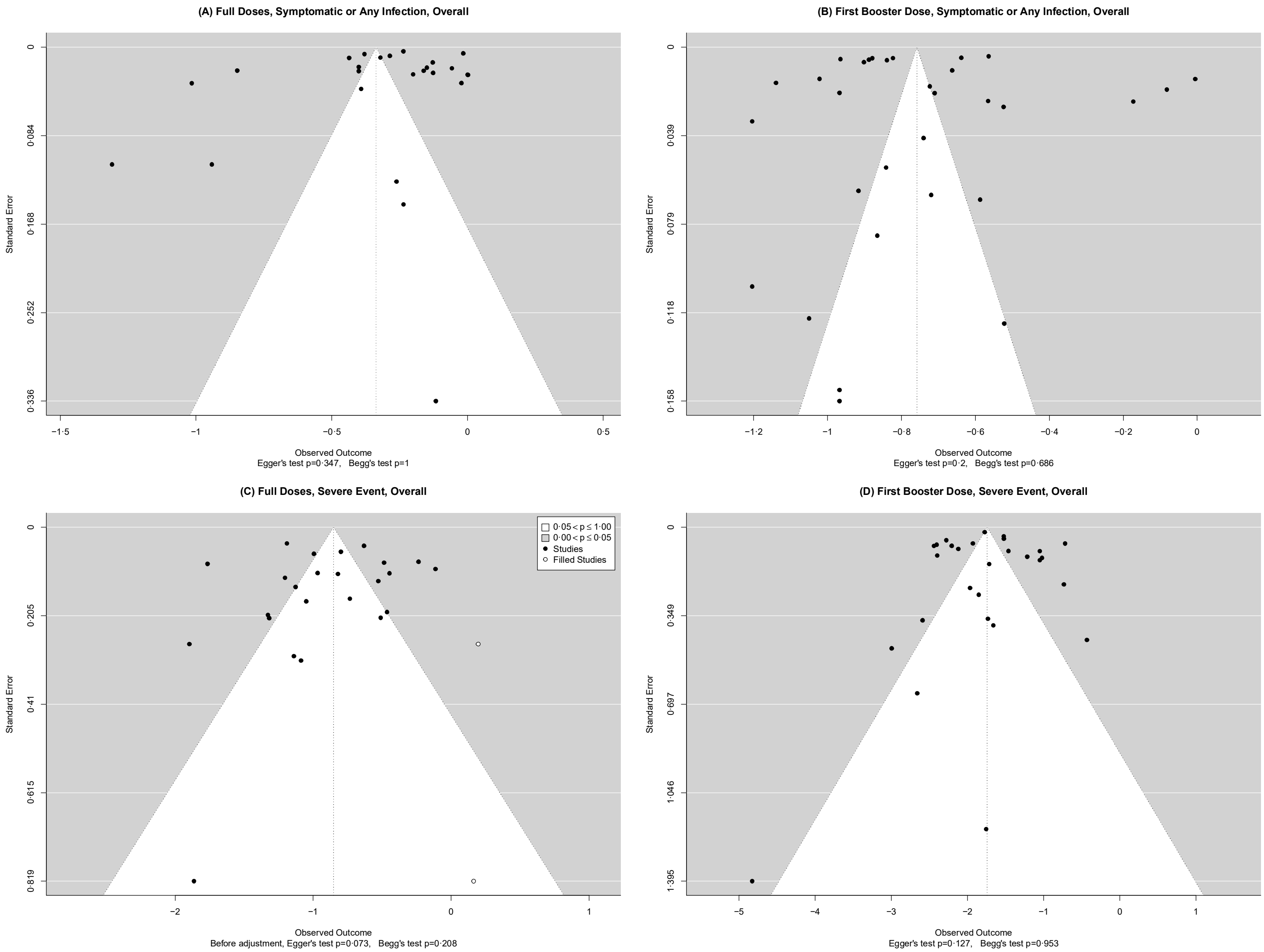

Supplementary Figure 8 Funnel plots for meta-analyses of overall VE estimates: (A) full doses against symptomatic infection or anyinfection; (B)first booster doses against symptomatic infection or any infection; (C) full doses against severe events; (D)first booster dosesagainst severe events.

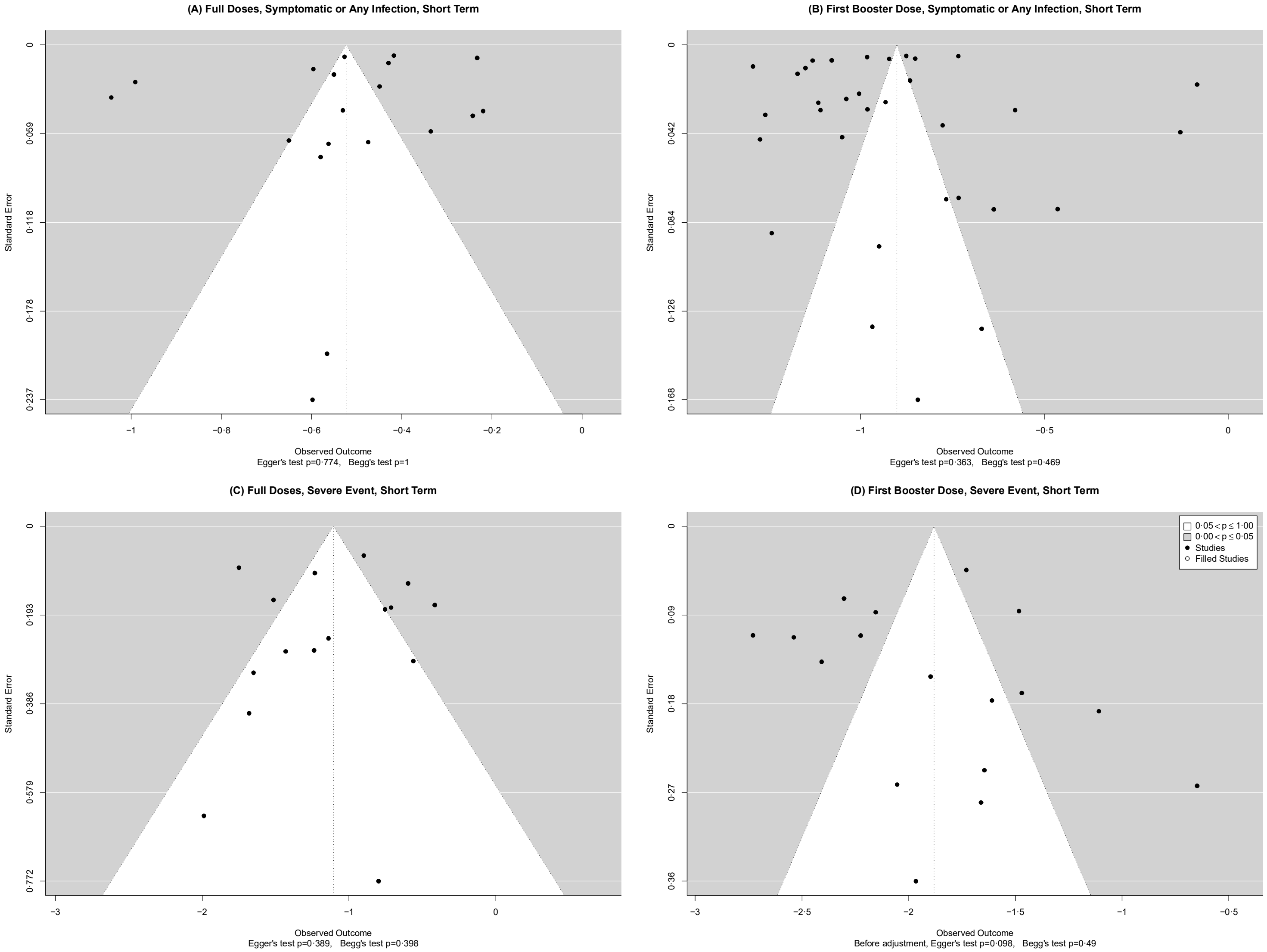

Supplementary Figure 9 Funnel Plots for meta-analyses of short-term VE estimates: (A) full doses against symptomatic infection oranyinfection; (B) first booster doses against symptomatic infection or any infection; (C) full doses against severeevents; (D) firstbooster doses against severe events

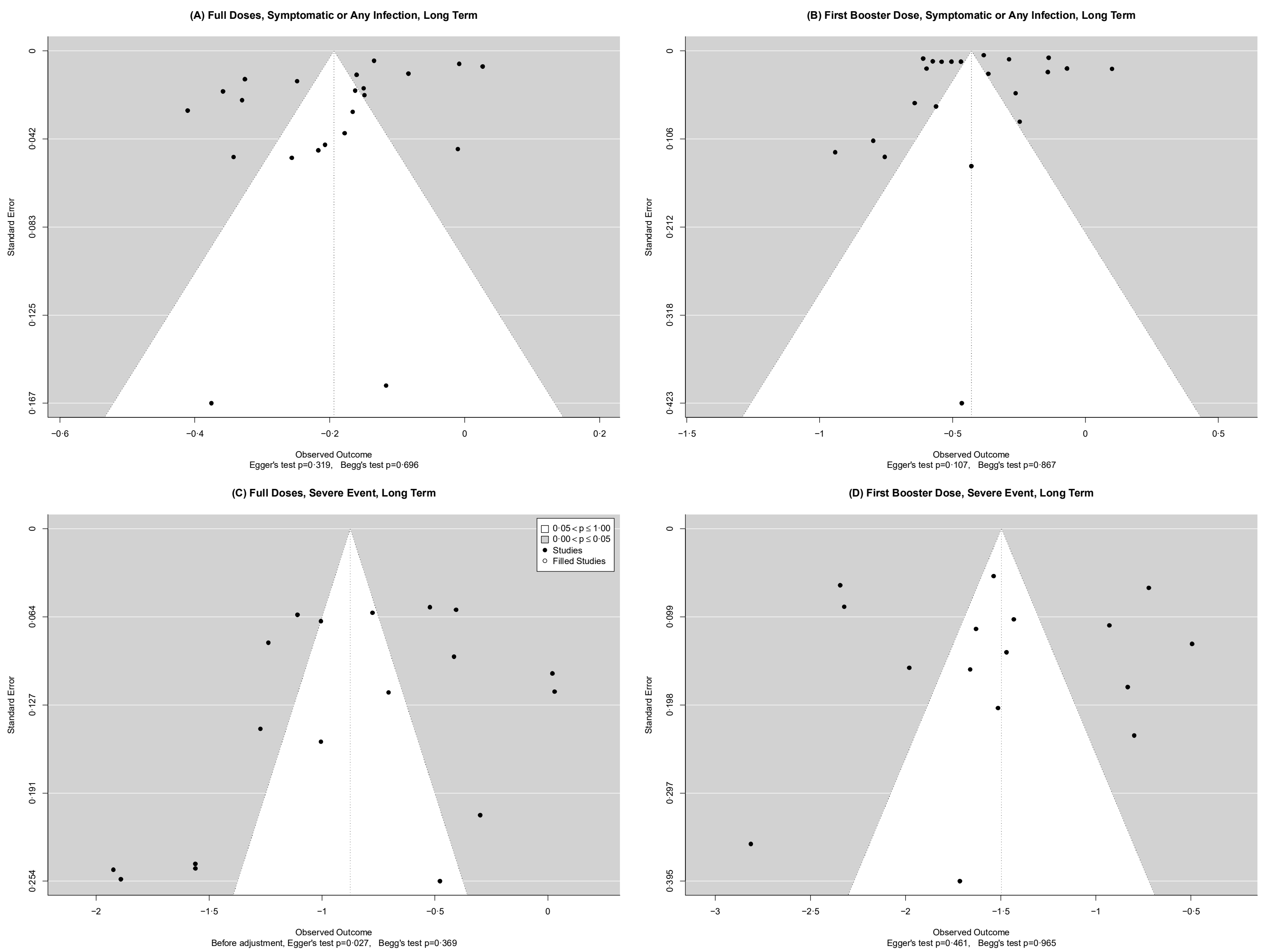

Supplementary Figure 10 Funnel Plots for meta-analyses of long-term VE estimates: (A) full doses against symptomatic infection or anyinfection; (B) first booster doses against symptomatic infection or any infection; (C) full doses against severe events; (D)firstbooster doses against severe events

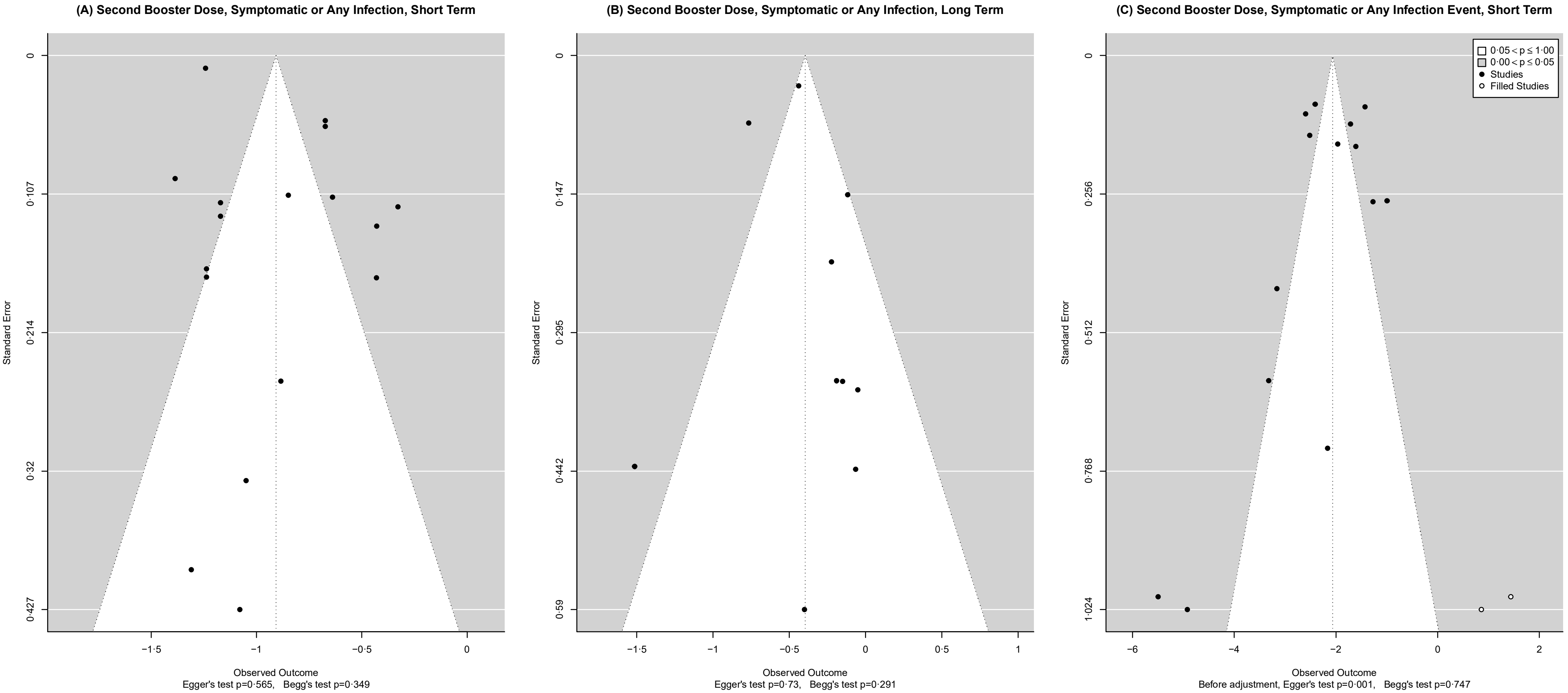

Supplementary Figure 11 Funnel Plots for meta-analyses of VE estimates of the second booster dose against: (A) symptomaticinfection or any infection in the short term; (B) symptomatic infection or anyinfection inthe long term; (C)severeevents in the short term. For the second booster, VE estimates were only available for adults.

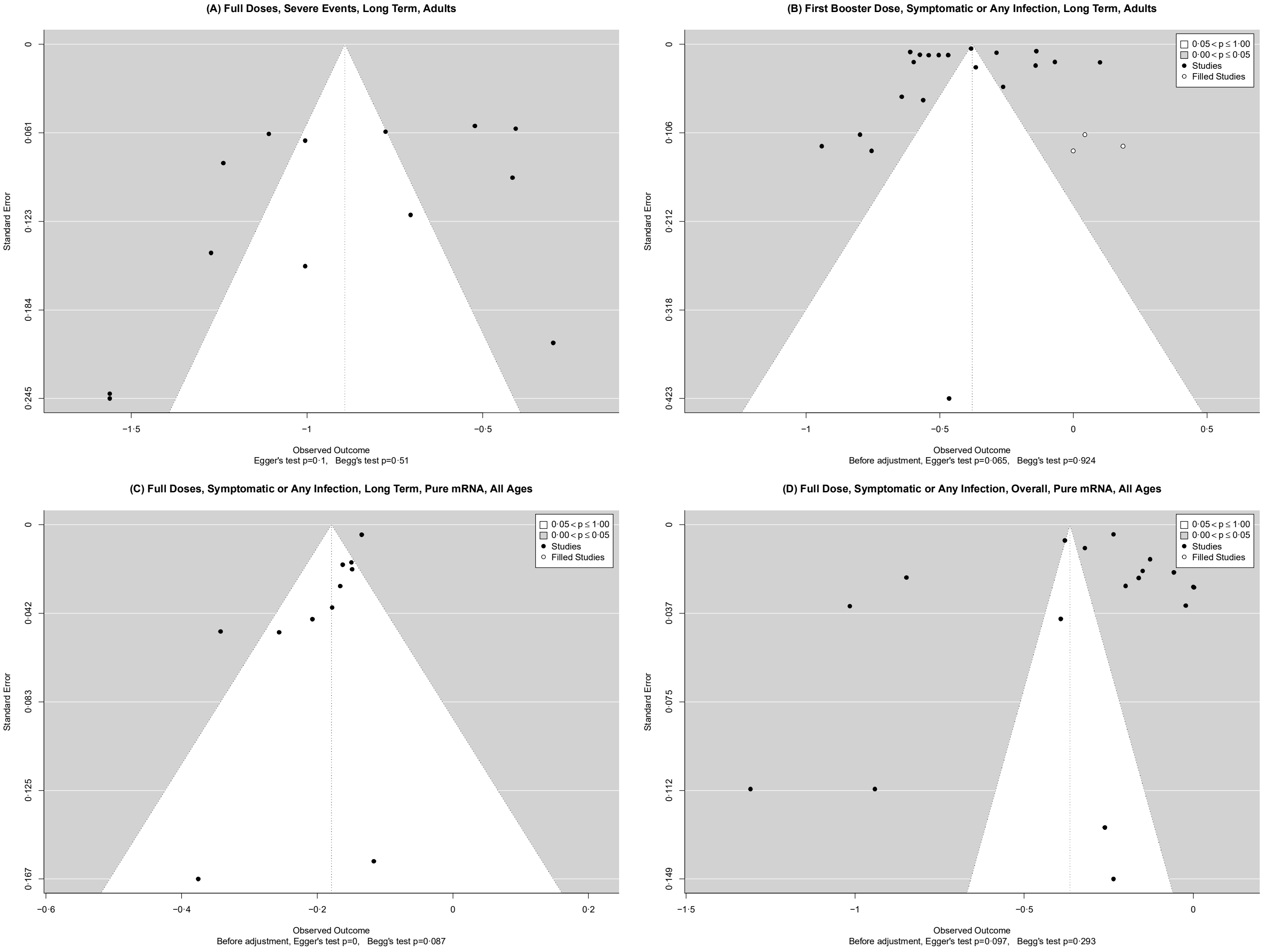

Supplementary Figure 12 Funnel plots for meta-analyses of VE estimates in subgroups defined by age group and vaccine type that showpublication bias (any p-value < 0·1)

Searching Strategies

Full Doses and Booster Search Strategy:
Search conducted time: June 27th, 2022
Publication time: November 26th, 2021, to June 27th, 2022, if a date can be specified; 2021 to 2022 if the year is the most specific scope.

Key words:
#1 (SARS-CoV-2) OR (COVID-19) OR (2019nCoV)
#2 (vaccine) OR (vaccination)
#3 (effectiveness) OR (efficacy)
#4 (test-negative) OR (case-control) OR (cohort study)
#5 Omicron
#6 (infected) OR (infection) OR (hospitalization) OR (hospital admission)

Note for #4, although our main interest is test-negative case-control design studies, we also searched for the other two possible study types to reduce the number of false negative results.

Database	Number of results
PubMed	82
Web of Science	23
Embase	89
Scopus	721
#1 AND #2 AND #3 AND #4 AND #5 AND #6 searched in all fields	

Cochrane library only supports up to 5 search terms

Database	Number of results
Cochrane Library	3
#1 AND #2 AND #3 AND #4 AND #5 searched in all fields	

Preprint databases used different searching rules and limitations

Database	Number of results
medRxiv	115
bioRxiv	6
for term "SARS-CoV-2 COVID-19 2019nCoV vaccine effectiveness test-negative case-control cohort study Omicron infection hospitalization" and posted between "November 26th, 2021 and June 27th, 2022"	

Google Scholar is a supplementary search source and can give many false positive results, so only the first ten pages of most relevant results will be screened.

Google Scholar	Top 100 out of 4,290
((SARS-CoV-2) OR (COVID-19) OR (2019nCoV)) AND ((vaccine) OR (vaccination)) AND ((effectiveness) OR (efficacy)) AND ((test-negative) OR (case-control) OR (cohort study)) AND (Omicron) AND ((infected) OR (infection) OR (hospitalization)	

Second Booster Search Strategy:
Search conducted time: Jan 08th, 2023
Publication time: November 26th, 2021, to Jan 08th, 2023, if a date can be specified; 2021 to 2023 if the year is the most specific scope.

Key words:
#1 (SARS-CoV-2) OR (COVID-19) OR (2019nCoV)
#2 (second booster) OR (additional booster) OR (fourth dose)
#3 (effectiveness) OR (efficacy)
#4 (test-negative) OR (case-control) OR (cohort study)
#5 Omicron
#6 (infected) OR (infection) OR (hospitalization) OR (hospital admission)

Note for #4, although our main interest is test-negative case-control design studies, we also searched for the other two possible study types to reduce the number of false negative results.

Database	Number of results
PubMed	56
Web of Science	22
Embase	55
Scopus	1015
#1 AND #2 AND #3 AND #4 AND #5 AND #6 searched in all fields	

Cochrane library only supports up to 5 search terms

Database	Number of results
Cochrane Library	9
#1 AND #2 AND #3 AND #4 AND #5 searched in all fields	

Preprint databases used different searching rules and limitations

Database	Number of results
medRxiv	149
bioRxiv	7
for term "SARS-CoV-2 COVID-19 2019nCoV second booster effectiveness test-negative case-control study Omicron infection hospitalization" and posted between "November 26th, 2021 and Sep 05th, 2022"	

Google Scholar is a supplementary search source and can give many false positive results, so only the first ten pages of most relevant results will be screened.

Google Scholar	Top 100 out of 4,110
((SARS-CoV-2) OR (COVID-19) OR (2019nCoV)) AND ((second booster) OR (additional booster) OR (fourth dose)) AND ((effectiveness) OR (efficacy)) AND ((test-negative) OR (case-control) OR (cohort study)) AND (Omicron) AND ((infected) OR (infection) OR (hospitalization)	
